# The influence of EPA and DHA on markers of inflammation in 3T3-L1 cells at different stages of cellular maturation

**DOI:** 10.1186/1476-511X-13-3

**Published:** 2014-01-03

**Authors:** Adam Prostek, Małgorzata Gajewska, Dariusz Kamola, Bożena Bałasińska

**Affiliations:** 1 Department of Physiological Sciences, Faculty of Veterinary Medicine, Warsaw University of Life Sciences (WULS-SGGW), Warsaw, Poland

**Keywords:** Adipocytes, Ageing, Inflammation, Obesity, EPA, DHA

## Abstract

**Background:**

EPA and DHA have been reported to have anti-obesity and anti-inflammatory properties. Recent studies revealed that these positive actions of n-3 PUFA at least partially are connected with their influence on metabolism and secretory functions of the adipose tissue. However, their impact on old adipocytes is still poorly understood. Therefore the aim of the present study was to evaluate the influence of EPA and DHA on markers of inflammation in 3T3-L1 cells at different stages of cellular maturation.

**Methods:**

Young, mature and old differentiated 3T3-L1 adipocytes were cultured for 48 h in the presence of 100 μM EPA, or 50 μM DHA complexed to albumin, whereas in control conditions only albumin was added to the medium. The Oil Red O staining was used to confirm adipocytes differentiation, and measure triglycerides content in cells. The concentration of adipokines (interleukin 6, adiponectin and leptin) in conditioned media was measured using mouse-specific ELISA kits.

**Results:**

The fat accumulation in 3T3-L1 adipocytes was positively correlated with their age; however, EPA and DHA did not affect lipid accumulation on any stage of maturation. EPA and DHA increased the concentration of secreted adiponectin when compared with control, but only in the case of young adipocytes (58% and 35%, respectively). Moreover, EPA supplementation increased interleukin 6 concentration in conditioned medium, while DHA exerted an opposite effect on all stages of cellular maturation. Furthermore, EPA treatment increased leptin release from young cells, while DHA did not affect the secretion of this adipokine. In mature 3T3-L1 adipocytes both experimental factors decreased synthesis of leptin; however, in old cells no impact of these PUFA was noted.

**Conclusions:**

In summary, age is an important determinant of fat accumulation in adipocytes and affects adipokines secretion by these cells. Moreover, the impact of investigated fatty acids: EPA and DHA on fat cells varies depending on the stage of maturation, and seems to be stronger in young cells than in mature and old ones. Docosahexaenoic acid exerts an anti-inflammatory action; however, on the basis of the obtained data it was not possible to determine whether eicosapentaenoic acid shows anti- or pro-inflammatory properties.

## Background

In recent years long chain polyunsaturated fatty acids n-3 (LC-PUFA n-3), such as eicosapentaenoic acid (EPA; C20:5; n-3) and docosahexaenoic acid (DHA; C22:6; n-3) are a subject of interest of many research centres around the world because of their beneficial effects on human and animal health. The most important positive actions of LC-PUFA n-3 are reduction of risk of cardiovascular diseases, cancer and dementia (Alzheimer’s disease) [1-3]. EPA and DHA have been reported to have protective effects in many types of chronic inflammatory conditions, such as: rheumatoid arthritis, asthma, Crohn’s disease and psoriasis [4]. The anti-inflammatory actions of n-3 PUFA are connected with their ability to decrease production of pro-inflammatory eicosanoids and cytokines [5]. Moreover, they are substrates for synthesis of other lipid mediators, such as anti-inflammatory protectins and resolvins [6].

Obesity is connected with increased immune activation and chronic state of low-grade inflammation in adipose tissue [7]. Low-grade inflammation in adipose tissue causes dysregulation of secretion of pro- and anti-inflammatory adipokines. Numerous recent studies have shown that secretion of proinflammatory cytokines (interleukin 6, TNF Alpha, MCP – 1, leptin) is increased in obesity; whereas, the concentration of anti-inflammatory cytokines (adiponectin, interleukin 10) is decreased [8-10]. The exact mechanism of inflammation in adipose tissue associated with obesity is still unclear. It is assumed that hypoxia [11] or endoplasmic reticulum stress (ER stress) [12] can play role in development of this anomaly.

The impact of EPA and DHA on adipose tissue metabolism, and their role in obesity prevention have gained an increasing attention in recent years [13]. It was observed that treatment of obese subjects with n-3 PUFA led to reduced circulating levels of pro-inflammatory cytokines and acute phase proteins [14]. In vitro and in vivo studies have revealed that these positive actions of n-3 PUFA at least partially are connected with their influence on metabolism and secretory functions of adipose tissue [15]. In most cases investigated fatty acids decreased secretion of pro-inflammatory adipokines, and increased synthesis of anti-inflammatory adipokines by adipose tissue cells.

Aging is associated with fat mass accretion [16]. Storing excess fat in adipocytes leads to their hypertrophy, and stimulates differentiation of new fat cells. However, it was demonstrated that the proliferation of preadipocytes and their ability to differentiate decreases with age in rats [17]. Furthermore, several recent studies have suggested that increased secretion of pro-inflammatory adipokines by adipocytes is positively correlated with cell aging [18,19] and their size [20]. These observations suggest that the metabolism and functions of mature and old adipocytes may play an important role in health status regulation, considering the fact that they constitute a large part of the fat tissue, especially in older individuals. The 3T3-L1 cells are a recognized in vitro model of adipogenesis. Results of many studies revealed that n-3 PUFA can affect metabolism and markers of inflammation in this type of cells. However, the effect of LC-PUFA n-3 on adipocytes on later stages of cellular maturation is still unknown. Therefore, the aim of the present study was to evaluate the influence of EPA and DHA on markers of inflammation in 3T3-L1 cells on different stages of cellular maturation.

## Results

### Effects of EPA and DHA on lipid accumulation and lipolysis

**Figure 1:**
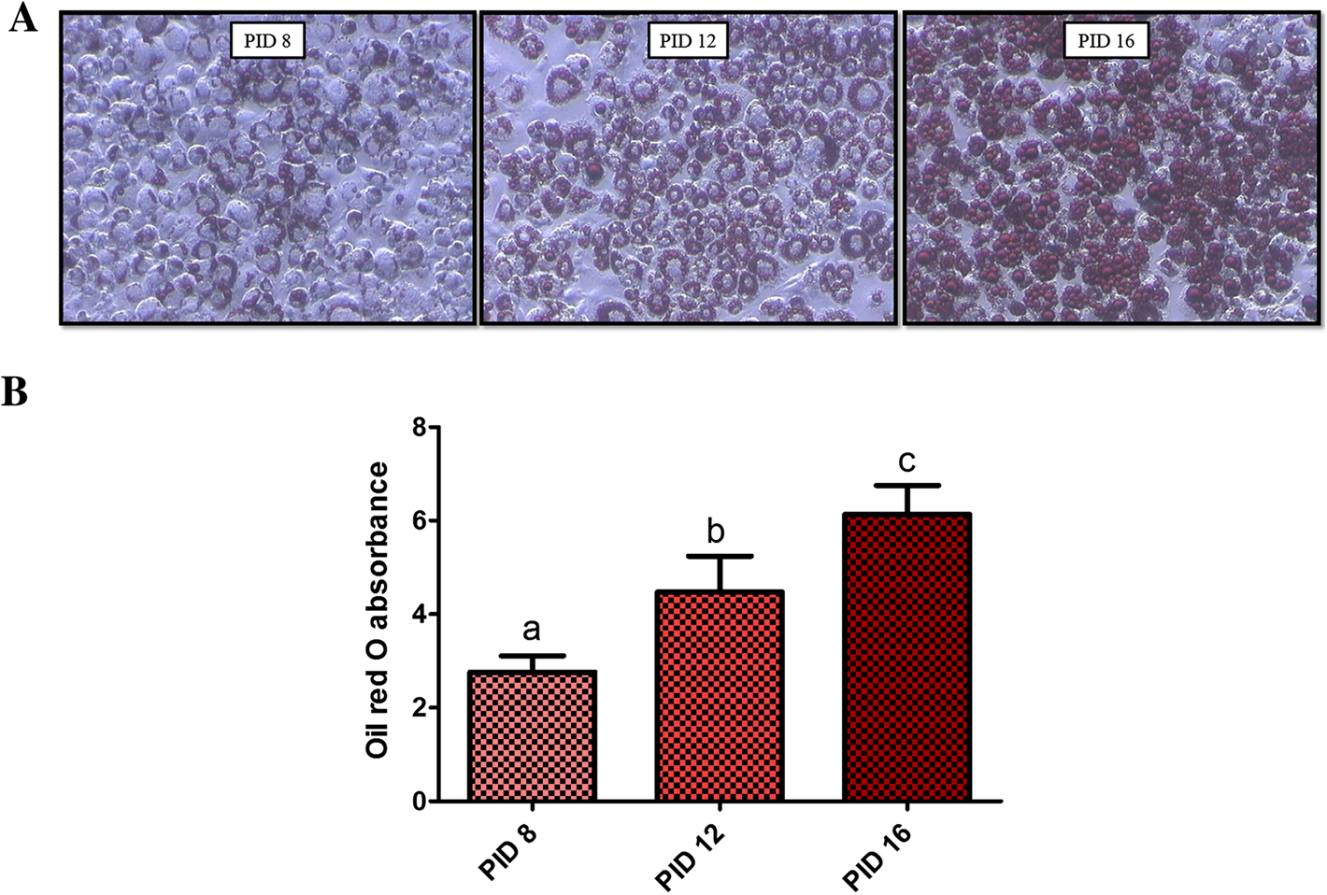
**Lipid accumulation in 3T3-L1 cells at different time of culture (days post induction of differentiation – PID). (A)** pictures from phase-contrast microscopy of cells stained with Oil red O on PID 8,12 and 16. **(B)** Absorbance of Oil red O extracted from 3T3-L1 cells on PID 8, 12 and 16.

Oil Red O was extracted from adipocytes and absorbance was measured to evaluate triglyceride contents in cells. The accumulation of lipids in differentiated 3T3-L1 cells was positively correlated with the day of culture (Figure 1). However, examined polyunsaturated fatty acids EPA and DHA did not affect lipid accumulation on any stage of maturation. Glycerol release was measured to determine the influence of EPA and DHA on the intensity of lipolysis in cultured adipocytes. Experimental factors had no impact on lipolysis in used concentrations (that constituted the highest concentrations showing no toxic effect in the cells, as evaluated in a preliminary study – data not shown).

### Effects of EPA and DHA on adipokines secretion by 3T3-L1 adipocytes

Age of cells (days post-induction of in vitro differentiation – PID) had a significant impact on secretion of adipokines. Concentration of pro-inflammatory cytokines was increased in conditioned medium of adipocytes from the 12th and 16th day of culture (PID) in comparison with the cells from the 8th day. Furthermore, the cells from the long-term culture secreted lower amounts of anti-inflammatory proteins. Time-dependent changes are presented in Table 1.

**Table 1:** Secretion of adipokines by 3T3-L1 cells during ageing (on days 8, 12, 16 post-induction of differentiation-PID)

	**PID 8**	**PID 12**	**PID 16**
Adiponectin concentration (ng/ml) ± SEM	1443.12 ± 31.52^a^	3870.25 ± 463.10^b^	2557.54 ± 104.90^c^
IL-6 concentration (pg/ml) ± SEM	33.41 ± 1.96^a^	122.30 ± 5.63^b^	738.54 ± 42.52^c^
Leptin concentration (pg/ml) ± SEM	91.33 ± 13.02^a^	562.40 ± 16.94^b^	436.21 ± 47.80^c^

Data are presented as means ± SEM, n = 3. Values in the same row that do not share the same superscript letter (eg. a, b or c) are significantly different according to one-way ANOVA (P < 0.05).

**Figure 2:**
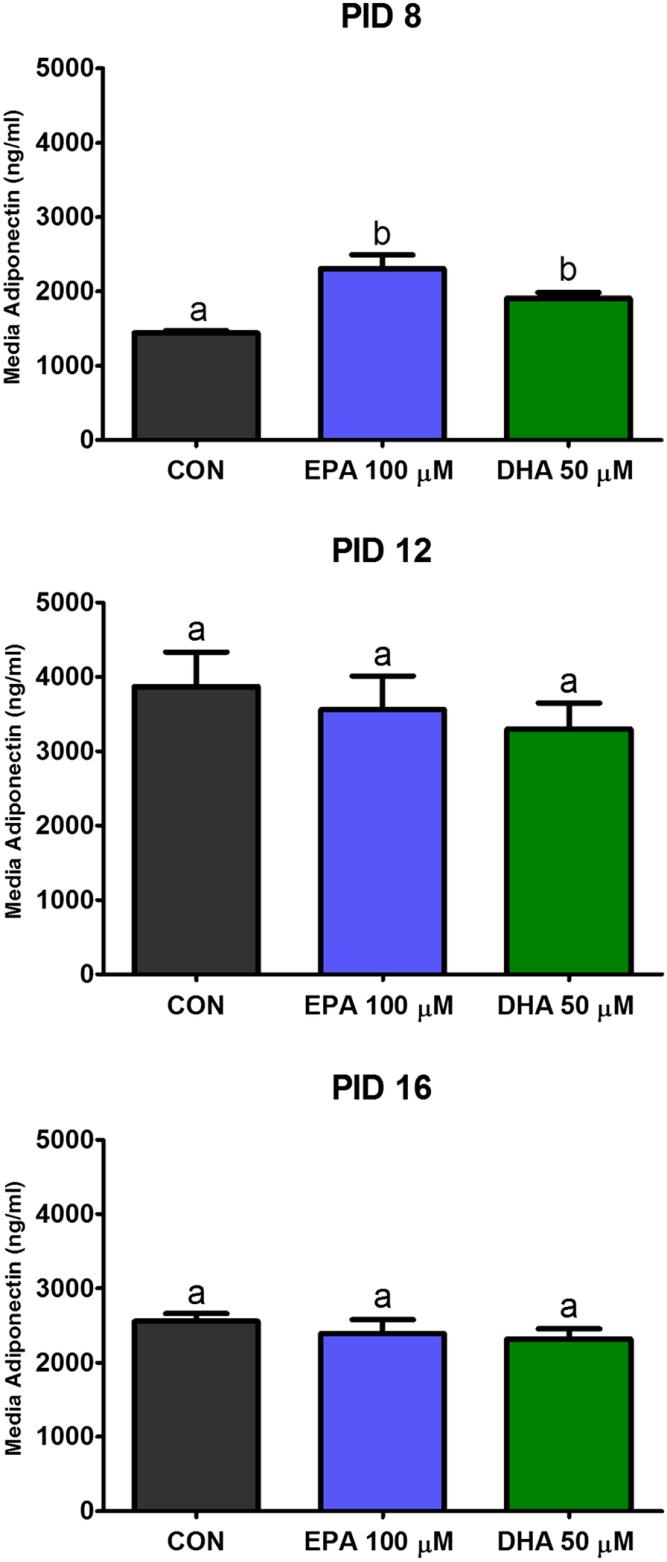
**Effects of EPA and DHA on secretion of adiponectin by 3T3-L1 adipocytes.** Concentration of adiponectin was measured in culture media collected on 8th, 12th, and 16th day post-induction of differentiation (PID 8,12 and 16). Data are presented as means ± SEM, n = 3. Means without a common letter differ, P < 0.05.

ELISA tests also revealed that both investigated polyunsaturated fatty acids increased the concentration of secreted adiponectin when compared with control (58% and 35%, respectively), but only on the early stage of maturation (PID 8). On PID 12 and 16 this effect was not observed (Figure 2).

**Figure 3:**
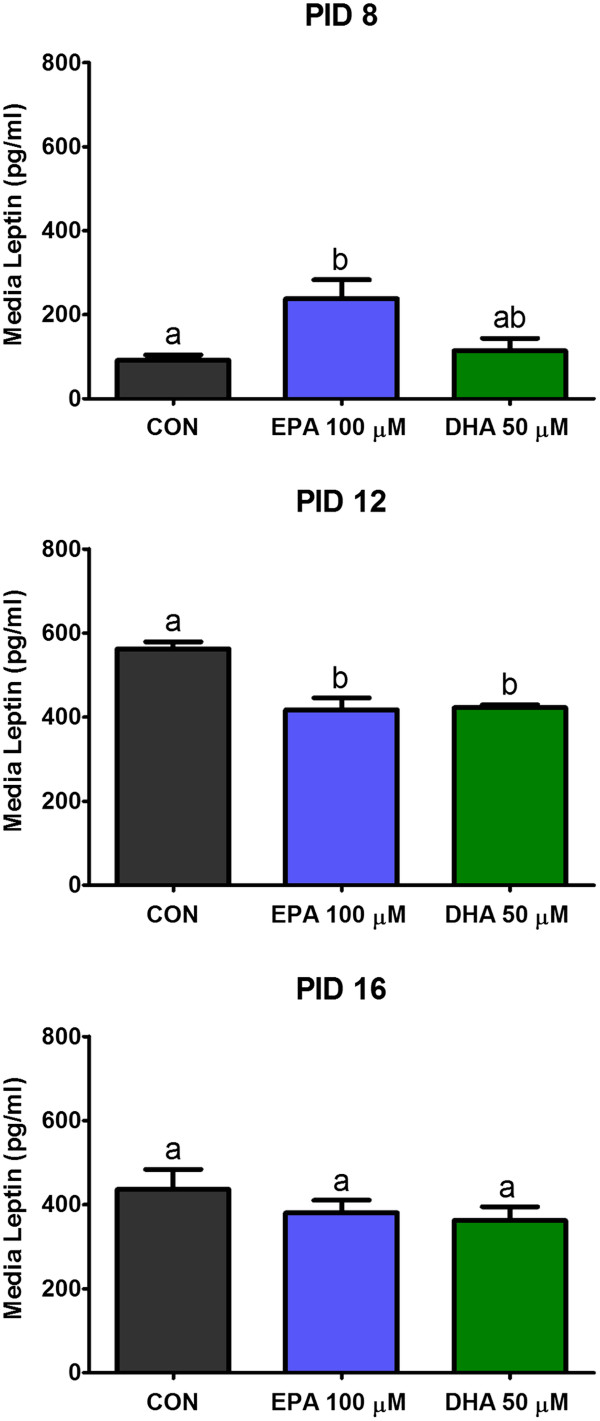
**Effects of EPA and DHA on secretion of leptin by 3T3-L1 adipocytes.** Concentration of leptin was measured in culture media collected on 8th, 12th, and 16th day post-induction of differentiation (PID 8,12 and 16). Data are presented as means ± SEM, n = 3. Means without a common letter differ, P < 0.05.

Moreover, as shown in Figure 3, EPA supplementation increased interleukin 6 concentration in conditioned medium, while DHA exerted an opposite effect. In the case of interleukin 6 similar impact of examined fatty acids was observed on PIDs 8,12 and 16.

**Figure 4:**
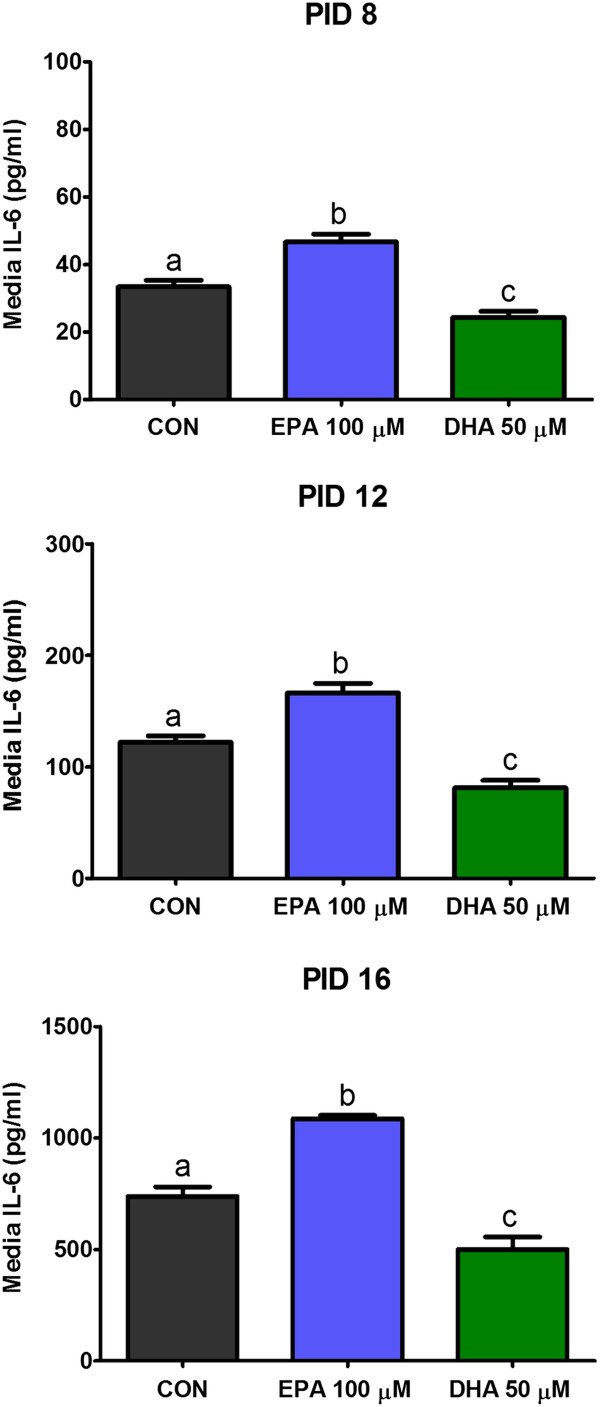
**Effects of EPA and DHA on secretion of IL-6 by 3T3-L1 adipocytes.** Concentration of IL-6 was measured in culture media collected on 8th, 12th, and 16th day post-induction of differentiation (PID 8,12 and 16). Data are presented as means ± SEM, n = 3. Means without a common letter differ, P < 0.05.

The influence of investigated n-3 PUFA on leptin secretion varied on different stages of maturation. On PID 8 EPA significantly increased leptin release form adipocytes, while DHA did not affect the secretion of this adipokine. On later days of culture (PID 12) both experimental factors decreased synthesis of leptin; however, on PID 16 no impact of these PUFA was noted (Figure 4).

### Cellular fatty acids content

**Figure 5:**
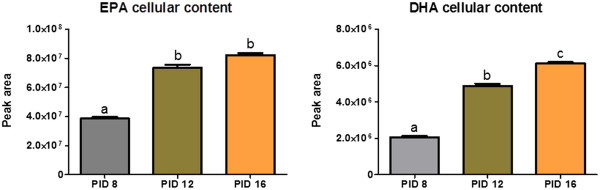
**Cellular content of EPA or DHA in 3T3-L1 cells during ageing.** Uptake of EPA or DHA by 3T3-L1 cells was measured on 8th, 12th, and 16th day post-induction of differentiation (PID 8,12 and 16). Data are presented as means ± SEM, n = 3. Means without a common letter differ, P < 0.05.

The content of investigated fatty acids: EPA and DHA in cells was quantified using GC-MS. The analysis showed that EPA and DHA uptake is age-dependent. The content of both fatty acids in cultured cells was the lowest on PID 8 and the highest on PID 16 (Figure 5). However, the content of examined EPA and DHA presented as the percentage of total fatty acids in cultured cells showed an opposite trend (Table 2).

**Table 2:** Investigated fatty acids content as the percentage of total fatty acids in 3T3-L1 cells during ageing

	**PID 8**	**PID 12**	**PID 16**
EPA content as the percentage of total fatty acids in EPA treated groups ± SEM	3.07 ± 0.15%^a^	3.97 ± 0.27%^b^	2.45 ± 0.15%^c^
DHA content as the percentage of total fatty acids in DHA treated groups ± SEM	0.36 ± 0.05%^a^	0.27 ± 0.02%^b^	0.16 ± 0.01%^c^

Data are presented as means ± SEM, n = 3. Values in the same row that do not share the same superscript letter (eg. a, b or c) are significantly different according to one-way ANOVA (P < 0.05).

## Discussion

In our experiment we hypothesized that the size of adipocytes is an important determinant of the adipokine secretion profiles. Accordingly, the large adipocytes released more pro-inflammatory and less anti-inflammatory adipokines than the lean fat cells. Studies have shown that large adipocytes are characteristic for adipose tissue of obese individuals. Moreover, obesity is more common in older people, in whom the ability of new adipocytes to differentiate is significantly decreased. There is a lack of information about inflammatory activity in old fat cells because in most of in vitro studies researchers use young adipocytes, in which the state of inflammation is induced by LPS treatment. In our study we used 3T3-L1 cells on different stages of growth and differentiation. Adipocytes on post-induction day 8 (PID 8) were considered as young, on PID 12 as mature, and on PID 16 as old. We noted time-dependent changes in control cells, which were maintained in standard medium without experimental factors. We observed that age of cultured cells was positively correlated with their fat accumulation. In addition, the presented results revealed significantly increased secretion of IL-6 (pro-inflammatory cytokine) by old adipocytes (PID 16) in comparison with young fat cells (PID 8). Furthermore, secretion of adiponectin (anti-inflammatory cytokine) was significantly decreased on PID 16 in comparison with PID 12. Thus, ageing of adipocytes can cause their large size and higher release of pro-inflammatory adipokines. Presented results are in agreement with a study by Zoico and co-workers, in which the authors also showed similar correlations [19]. These observations suggest that differentiated 3T3-L1 cells on the later stages of maturation can constitute a good experimental model for studying obesity and inflammation in adipose tissue.

Our study also aimed at evaluating the influence of EPA and DHA, which are regarded as PUFA with potential anti-obesity and anti-inflammatory properties, on fat cells secretory activities in relation to their age (days of culture). To our knowledge this is the first study investigating the effect of EPA and DHA during ageing of adipocytes. Our experiment did not reveal any differences in the intensity of lipolysis (glycerol release) and fat accumulation (Oil red O absorbance) between adipocytes treated with EPA, DHA and control cells. These results are contrary to previous findings showing that both EPA and DHA may increase lipolysis in differentiated 3T3-L1 cells [21-23]. However, it is hard to compare our data with the cited studies, in which the doses of evaluated fatty acids were higher, and the time of exposure was shorter. We used higher concentrations of EPA (300 μmol/l) and DHA (100, 200 μmol/l) in our preliminary studies, but these doses caused increased cell death (data not shown). Our observations indicate that the anti-obesity properties of n-3 PUFA are not connected with their influence on lipolysis in adipocytes, but possibly with induction of apoptosis in these cells.

Although we did not observe any effect of EPA and DHA on lipolysis and fat accumulation, we noted their significant role in secretion of adipokines by adipocytes on different stages of maturation. First adipokine investigated in our study was adiponectin, belongs to the anti-inflammatory adipokines secreted by adipose tissue, mainly by adipocytes. In organisms this protein is involved in i.e. glucose metabolism, fatty acids β-oxidation and insulin sensitivity [24]. Numerous studies have shown that in obese humans and animals with insulin resistance, concentration of plasma adiponectin is significantly decreased. Moreover, increased endogenous production of this hormone improves insulin sensitivity [25]. In the last few years, several studies have suggested that n-3 PUFA are able to decrease insulin resistance connected with obesity, possibly through their stimulatory effect on adiponectin secretion. However, the influence of examined fatty acids on synthesis of this protein by adipocytes is still unclear. Recent data from in vivo experiments on rodents seem to confirm the abovementioned thesis. On the other hand, in vitro studies provided contrary information on this subject. Kalupahana and co-workers performed an experiment on mice, as well as 3T3-L1 cell line, and revealed that diet supplementation with EPA improved adiponectin levels in blood of obese mice, but the authors did not observe any influence of EPA (150 μM) treatment on adiponectin release by differentiated 3T3-L1 cells [26]. Findings of another group showed that both EPA and DHA (250 μM) were able to decrease the expression of apM1 gene, which encodes adiponectin [27]. Our study demonstrated that investigated n-3 PUFA (EPA 100 μM, DHA 50 μM) increased adiponectin secretion by 3T3-L1 adipocytes, but only on the early stage of maturation (PID 8). These results are partially in agreement with the study by Oster and co-workers, who observed similar effects of EPA and DHA in young cells [28]. However, our experiment showed that the stimulatory activity of both PUFA on adiponectin secretion by adipocytes was not sustained on the later days of culture (PID 12 and PID 16). Different findings of in vitro experiments performed on young fat cells could be explained by various doses used in both studies. Oster et. al [28] suggested that high concentrations of EPA and DHA can cause more intense lipid peroxidation in adipocytes, and development of oxidative stress associated with reduction of adiponectin gene expression.

We also examined the concentration of leptin released by 3T3-L1 adipocytes. White adipose tissue is the main source of this hormone in the organism, but it is also secreted by stomach, brain and bone morrow [29]. This adipokine has a systemic action and is engaged in the regulation of food intake, energy expenditure, body fat storage and insulin signalling [30]. Moreover, high blood concentrations of leptin are strongly correlated with obesity, and it was observed that these concentrations decrease with weight loss. Several reports, based on in vitro and in vivo experiments, revealed that n-3 PUFA are able to modulate leptin secretion by adipocytes. In our study we demonstrated a stimulatory effect of EPA supplementation on this hormone release by young (PID 8) 3T3-L1 adipocytes, but at this stage DHA did not show any impact. In the later period of cell culture we observed that both EPA and DHA significantly decreased secretion of leptin by cells on PID 12, but this effect was not noted on PID 16. The stimulation of leptin secretion by EPA observed on PID 8 was in agreement with other in vitro studies, showing that this fatty acid is able to increase leptin gene expression and leptin secretion in 3T3-L1 cells, as well as primary cultures of rat adipocytes [30,31]. In contrast, Reseland and co-workers reported that EPA and DHA decreased leptin mRNA expression in vitro and in vivo [32]. However, the results obtained by Reseland group are incomparable with our findings, because they used much higher doses of n-3 PUFA (500 μM). Other in vivo studies also showed that long-term diet supplementation with n-3 PUFA, resulted in decreased plasma leptin concentrations in rats and mice [32,33]. This inhibitory effect observed in vivo is usually correlated with the anti-obesity influence of n-3 PUFA. To our knowledge, this is the first report in which the influence of EPA and DHA on leptin secretion has been investigated in 3T3-L1 adipocytes on different stages of cellular maturation. Our observations showing the opposite effects of these fatty acids on leptin secretion between PID 8 and PID 12, and no impact on PID 16 indicate that adipocytes reaction and sensitivity to EPA and DHA are age-dependent.

We also examined the influence of n-3 PUFA on secretion of interleukin 6 (IL-6) by 3T3-L1 cells during their aging. IL-6 is a pro-inflammatory cytokine secreted by monocytes, macrophages and fat cells. Studies have shown that the level of blood circulating IL-6 is significantly increased in obese subjects [34]. Adipose tissue is a source of approximately 30% of this cytokine in the organism [35]. IL-6 participates in the development of insulin resistance via different mechanisms. Firstly, this adipokine decreases adiponectin secretion by adipose tissue. Moreover, it directly affects the insulin signal transduction pathway in hepatocytes [36]. The mechanism of this action is not completely understood, but it is believed that SOCS3 protein (suppressor of cytokine signal 3) may be involved. SOCS3 probably inhibits the insulin-dependent autophosphorylation of insulin receptor [37]. Presented results showed that secretion of this pro-inflammatory adipokine by fat cells was strongly connected with their age, and was the highest in old adipocytes (PID 16). n-3 PUFA have been reported to have anti-inflammatory properties. Surprisingly, in our study we found that EPA supplementation increased IL-6 concentration in conditioned medium, while DHA exerted an opposite effect. Similar impact of investigated fatty acids was observed at all stages of cellular maturation. Contrary results were obtained by other research groups working with young adipocytes. Siriwardhana and co-workers observed that EPA treatment (200 μM) increased secretion of IL-6 in differentiated 3T3-L1 cells [38]. On the other hand, Kalupahana and co-workers found that 150 μM concentration of EPA in incubation medium inhibited IL-6 release by adipocytes [26]. These differences are hard to explain because in all experiments the time of treatment was the same, and the reaction of 3T3-L1 cells to n-3 PUFA treatment was not dose-dependent. These discrepancies show that this topic needs further investigation in the future. However, the results of our study indicate that EPA and DHA are able to modulate secretion of IL-6 not only in young cells but also in ageing adipocytes.

In addition, we examined the content of polyunsaturated fatty acids: EPA and DHA in cultured cells. The results obtained revealed that the uptake of investigated fatty acids increased with age. On the other hand, the percentage of EPA and DHA in relation to the total quantity of fatty acids decreased with age in most cases. The second observation correlated with age-dependant differences in secretion of examined adipokines and could at least partially explain the weaker effect of EPA and DHA on endocrine functions of old cells in comparison with young and mature adipocytes.

## Conclusions

Age is an important determinant of fat accumulation in adipocytes and affects adipokines secretion by this type of cells. Older adipocytes are more filled with fat, and release more pro-inflammatory factors. These observations suggest that differentiated 3T3-L1 cells on later stages of maturation can serve as a good experimental model for studying obesity and inflammation in adipose tissue. EPA and DHA did not affect the accumulation of fat and lipolysis in 3T3-L1 cells, but significantly altered secretion of adipokines. The influence of these fatty acids on adipokines release by 3T3-L1 cells varied at different stages of cellular maturation. Action of DHA seems to be anti-inflammatory; however, on the basis of the data obtained it was not possible to determine whether EPA exerts anti- or pro-inflammatory properties. The effects of evaluated fatty acids on endocrine function of adipocytes were stronger in young cells than in mature and old ones, which could be related to lower percentage content of n-3 PUFA in total fatty acids in old cells when compared with young and mature cells.

## Methods

### Cell culture

3T3-L1 mouse embryo fibroblasts were purchased from American Type Culture Collection (Rockville, MD USA) and cultured in humidified atmosphere of 5% CO_2_, 95% air at 37°C. The cells were maintained in a growth medium containing the following components: Dulbecco’s modified Eagle’s medium (Sigma) with high glucose, 10% newborn calf serum (NBCS, obtained from Invitrogen) and 1% penicillin-streptomycin (P/S, Sigma). Two days after the cells reached confluence (post-induction day 0), differentiation to adipocytes was initiated using differentiation medium supplemented with: 1 μM dexamethasone (Sigma), 0.5 mM isobutylmethylxanthine (Sigma) and 10 μg/ml insulin (Sigma). In differentiation medium 10% addition of NBCS was also replaced with 10% fetal bovine serum (FBS, GIBCO). After 2 days (post-induction day 2) fresh medium containing only insulin was added for further 2 days. On post-induction day 4 medium was replaced with DMEM supplemented with 10% FBS and antibiotics. The cells were maintained in this type of medium until fatty acid treatment. Medium was change every two days.

### Fatty acids treatments

**Figure 6:**
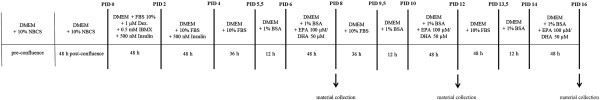
Schematic presentation of the experiment with marked days in which material for analyses was collected.

On post-induction days (PID) 6, 10 and 14 differentiated 3T3-L1 adipocytes were cultured for 48 h in the presence of 100 μM EPA, or 50 μM DHA complexed to albumin; whereas, in control conditions only albumin was added to the medium. Experimental media consisted of serum-free DMEM, 1% FA-free bovine serum albumin (BSA), EPA/DHA/albumin and antibiotics. Every fatty acid treatment was preceded by 12-hour starvation in serum-free DMEM supplemented with 1% FA-free BSA and antibiotics. On PID 8, 12 and 16 conditioned media were collected and stored at – 80°C until further analyses. The scheme of the experiment is presented in Figure 6.

### Oil Red O staining

The Oil Red O staining was used to confirm adipocytes differentiation and measure triglycerides content in cells. After appropriate treatment conditioned media were collected, adipocytes were washed with cold phosphate-buffered saline (PBS; pH 7.4) and fixed with 4% paraformaldehyde solution in PBS for 30 minutes. Subsequently, cells were washed twice in PBS and stained in Oil Red O working solution (stock – water, 3:2) for 30 minutes. Then Oil Red O was discarded and cells were washed with water 4 times. Images of stained cells were taken using phase contrast microscope (Olympus IX-70 microscope, Optical Co., Hamburg, Germany). Additionally, a quantitative measurement of triglycerides was performed. Oil Red O was extracted from adipocytes by isopropanol and absorbance was spectrophotometrically measured at 500 nm.

### Preparation of fatty acid methyl esters

Cells were collected on PID 8, 12 and 16, and fatty acids were extracted by a mixture of chloroform and methanol (2:1). Chloroform – methanol was added to cell pellets, and the samples were vortex-mixed for 15 min, and left to form a bilayer. The aqueous phase was discarded, and the organic layer was evaporated using nitrogen. Two milliliters of 0.5 M NaOH in methanol was added, and the samples were incubated for 1 hour at 85°C. Then the samples were cooled on ice, and unsaponifiable lipids were removed by extraction with hexane. The residue was acidified to pH 2 with 4 M HCl, and the free fatty acids were extracted with hexane. The solvent was evaporated under nitrogen, and fatty acids were esterified with 2 M HCl in methanol for 45 minutes at 85°C. When the samples were cooled down, fatty acid methyl esters (FAME) were extracted with hexane.

### GC-MS analysis

A Perkin Elmer model Clarus 500 selective mass detector coupled to a model Clarus 500 GC was used. FAME were separated on the GC with a ZB-5MSi column (30 m × 0.25 mm ID ×1.00 μm film thickness, Phenomenex USA) with the following conditions: helium flow rate, 1 mL/min; initial oven temperature, 50°C, with increase at 15°C/min to 280°C, and increase at 3°C/min to a final temperature of 300°C and held for 3 minutes. The expected retention times for EPA and DHA were: 20.35 and 22.34 min, respectively. Mass spectra of fatty acids were acquired using electron impact ionization in total ions current mode.

### Glycerol measurements

Glycerol released into the media was quantified by commercial glycerol test (Randox, cat. no. GY105) according to manufacturer’s instructions.

### Adipokines analysis

The concentration of adipokines in conditioned media was measured using mouse-specific ELISA kits. The following adipokines were quantified: interleukin 6 (Diaclone, cat. no. 860.020.096), adiponectin (Biovendor, cat. no. RD293023100R) and leptin (Mediagnost, cat. no. E06). All analysis were performed according to manufacturer’s instructions.

### Statistical analysis

All results are expressed as means ± SEM. Data were analyzed by GraphPad Prism^TM^ version 5.00 (GraphPad Software, Inc., USA). The statistical significance of differences between groups was determined by one-way analysis of variance (One-way ANOVA) and Tukey’s multiple-comparison post-test. The results were considered to be significant when the value of P was <0.05.

## Competing interests

The authors declare that they have no competing interests.

## Authors’ contributions

AP and BB designed the study. AP and MG performed the experiments. AP and BB analyzed the data and wrote the first draft of manuscript. MG and DK revised the manuscript. All authors read and approved the final manuscript.
